# Ornamental bromeliads of Miami-Dade County, Florida are important breeding sites for *Aedes aegypti* (Diptera: Culicidae)

**DOI:** 10.1186/s13071-018-2866-9

**Published:** 2018-05-17

**Authors:** André B. B. Wilke, Chalmers Vasquez, Paul J. Mauriello, John C. Beier

**Affiliations:** 1 Department of Public Health Sciences, Miller School of Medicine, University of Miami, Miami, FL USA; 2 Miami-Dade County Mosquito Control Division, Miami, FL USA; 3 Miami-Dade County Department of Solid Waste Management, Miami, FL USA

**Keywords:** Vector ecology, Urbanization, Bromeliaceae, Zika virus

## Abstract

**Background:**

A major public health concern is the emergence and geographical spread of vector-borne diseases such as Zika and yellow fever. Ornamental bromeliads retaining water in their leaf axils represent potential breeding sites for mosquitoes. As the role of ornamental bromeliads in breeding *Aedes aegypti* in Miami-Dade County, Florida is unknown, we hypothesize that ornamental bromeliads are important breeding sites for *Ae. aegypti*. Our objective was to survey bromeliads in areas with high densities of adult *Ae. aegypti*, including those with 2016 local transmission of Zika virus.

**Methods:**

Ornamental bromeliads were surveyed for the presence of immature mosquitoes at 51 locations of Miami-Dade County, Florida. Bromeliads were sampled for the presence of immature stages of mosquitoes, their reservoirs were drained and screened for the presence of immature mosquitoes. Immature mosquitoes were stored in plastic containers and preserved in 70% ethanol until morphological identification. Biodiversity of species assemblages was assessed by Shannon’s and Simpson’s indices, and individual rarefaction curves and plots of cumulative abundance, Shannon’s index and evenness profiles.

**Results:**

Ornamental bromeliads were present in all surveyed areas, yielding a total of 765 immature mosquitoes, comprising five taxonomic units: *Ae. aegypti*, *Wyeomyia mitchellii*, *Wyeomyia vanduzeei*, *Culex quinquefasciatus* and *Culex biscaynensis*. The biodiversity indices point to a low diversity scenario with a highly dominant species, *Ae. aegypti*.

**Discussion:**

Our findings suggest that ornamental bromeliads are contributing for the proliferation of *Ae. aegypti* in the County of Miami-Dade, which may indicate a shift in the paradigm of usage of bromeliads as breeding sites, highlighting that ornamental phytotelmata bromeliads are to be considered in future vector-control strategies to control Zika and other arboviruses.

**Electronic supplementary material:**

The online version of this article (10.1186/s13071-018-2866-9) contains supplementary material, which is available to authorized users.

## Background

Vector-borne diseases are an increasing public health concern, they are spreading to new areas due to urbanization, human movement and global warming [1–3]. Anthropogenic alterations in the environment are positively associated with the decrease in richness and increase in abundance of selected species of vector mosquitoes that are adapted to live in the urban environment, significantly impacting the risk of vector-borne disease transmission [4–6].

Substantial efforts have been made to control *Aedes aegypti* mosquitoes, a highly invasive species with worldwide distribution and highly adapted to urban environments. It is often positively associated with human population densities, laying eggs in artificial breeding sites and blood-feeding in human hosts. Moreover, *Ae. aegypti* is the main vector for dengue fever (DENV), chikungunya (CHYKV), yellow fever (YFV) and Zika (ZIKV) viruses [6–10]. Recently, it was responsible for the spreading of ZIKV in the Americas resulting in more than 700,000 cases, and subsequent introduction in the USA, resulting in hundreds of reported cases of local transmission in the County of Miami-Dade, Florida [11, 12].

The (re-)emergence and spreading of vector-borne diseases such as YFV and ZIKV is inevitable [13–15], and according to the World Health Organization (WHO) integrated vector management (IVM) is the most effective and sustainable strategy for the prevention of vector-borne diseases. IVM consists of scientific driven decision-making process to increase the effectiveness of the available resources for vector control, including traditional and new strategies that can be adapted to the target species such as *Ae. aegypti*, *Anopheles gambiae* and *Culex quinquefasciatus* [16–18]. Consisting of the use of both chemical and non-chemical control strategies, the IVM prioritize active surveillance of vector-mosquitoes, removal of breeding sites and campaigns for population consciousness [19]. Moreover, human behavior is a key driver for the population dynamics of *Ae. aegypti*, structural man-made alterations in the urban landscape commonly found around households are excellent breeding sites to their immatures [20–22].

Plants such as bromeliads are a popular choice for landscaping projects, widely found throughout urban areas since they are very resilient and do not need much care. Bromeliads (*Bromeliaceae*) are a family of Neotropical plants, composed by around 50 genera and more than three thousand species, they are capable of absorbing nutrients from the water retained in their leaves axils and central tank, sheltering several species of insects, some of them with epidemiological relevance [23]. Anopheline species, mostly from the subgenus *Kerteszia*, have phytotelmata bromeliads as primary breeding sites, rendering these plants important for the epidemiology of malaria [24, 25].

Ornamental bromeliads comprise a complex scenario in which the composition of mosquito species vary locally, often affected by heavy rainfall and seasonality. Furthermore, the role of ornamental bromeliads in breeding *Ae. aegypti* has been considered unimportant by several studies [26–28]. However, intense selective pressures present in urbanized areas may be modulating behavioral changes in *Ae. aegypti*. Previous studies found that urban populations are laying eggs in brackish water and sewage, indicating adaptations to new breeding sites [29, 30].

The Miami-Dade County Mosquito Control Division has greatly intensified vector-mosquito suppression efforts since the ZIKV outbreak in 2016. This includes considering the potential risk of ornamental bromeliads breeding vector mosquitoes, applying insecticides, removing plants when necessary, in addition to issuing brochures to notify and educate the public (Additional file 1: Figure S1). However, scientific evidence whether *Ae. aegypti* and other vector mosquitoes are breeding in ornamental bromeliads is lacking for Miami-Dade County, as well as how vector mosquito population dynamics has been driven by the presence of these plants.

An understanding of how vector mosquitoes are adapting locally to ornamental bromeliads is needed by Miami-Dade Mosquito Control Division to locally tailor surveillance-response programs. Taking this into account, our hypothesis is that ornamental bromeliads commonly used for landscaping purposes in Miami-Dade County, Florida are important breeding sites for *Ae. aegypti*. Therefore, our objective was to survey bromeliads in urbanized areas with high densities of *Ae. aegypti*, including those with previous local transmission of ZIKV.

## Methods

**Fig. 1:**
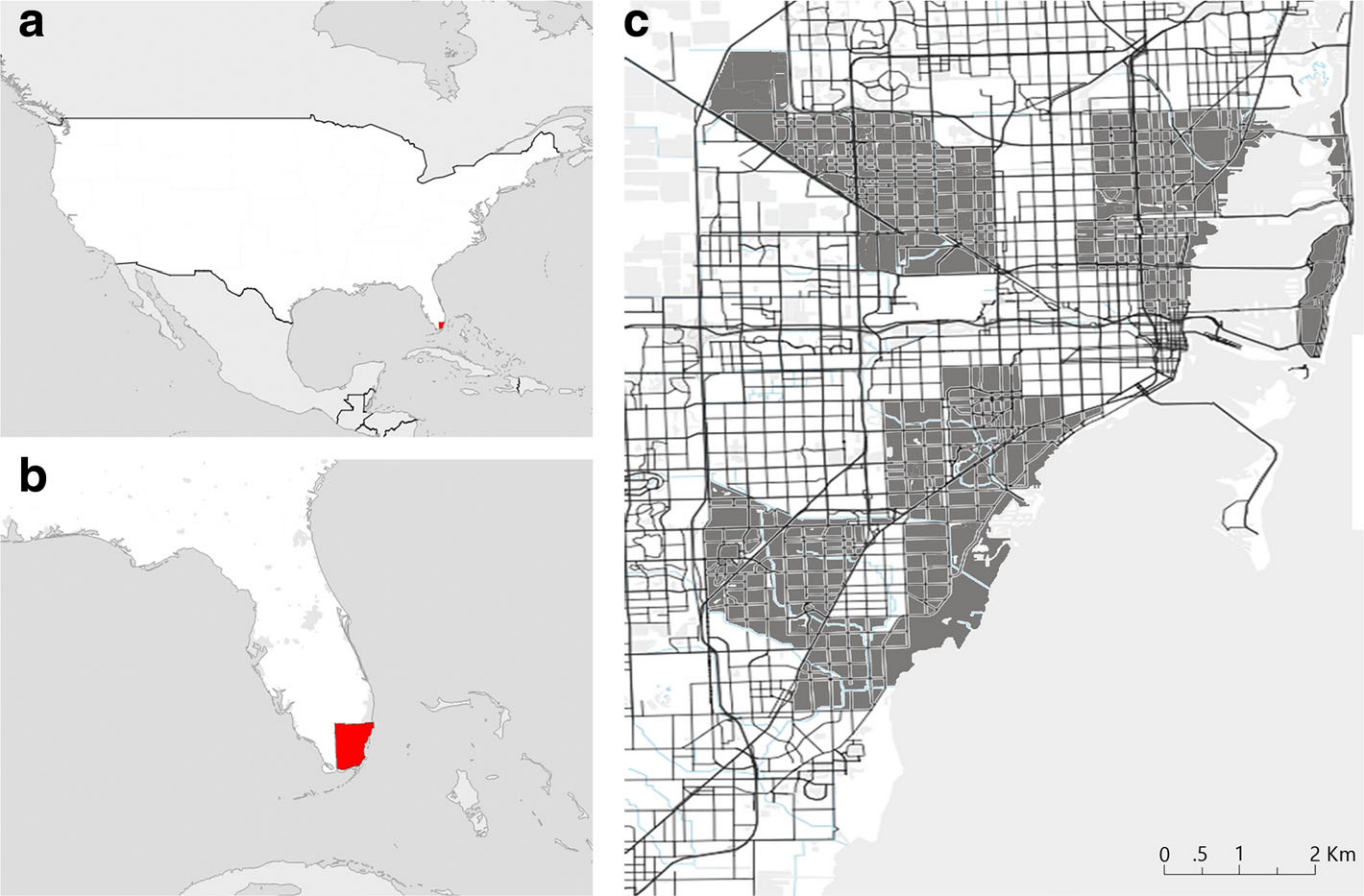
Maps showing surveyed areas for immature mosquitoes breeding in ornamental bromeliads in the County of Miami-Dade. **a** North America. **b** Southeast United States. **c** Miami-Dade County

The 51 study locations were selected for the survey-based bromeliad study because of their unique socioeconomic and environmental characteristics including normalized difference vegetation index (NDVI), income and the risk of vector-borne disease transmission as previously described [31–33]. This allowed us to capture variation among the 11 neighborhoods and 18 ZIP Codes in which the 51 locations were situated in Miami-Dade County, Florida as well as the distinctive conditions for mosquito proliferation and high densities of adult *Ae. aegypti*. Three of the 11 study locations were neighborhoods with 2016 local transmission of Zika virus (South Beach, Wynwood and Little River) (Fig. 1). Ornamental bromeliads were surveyed once a week for five weeks between June and July 2017 for the presence of immature mosquitoes.

Aiming for a wide coverage area, all bromeliad plants in each area were sampled for the presence of immature stages of mosquitoes. The sampling effort was standardized for all collections, consisting of draining bromeliads’ water reservoir tanks with the aid of manual plastic pumps (turkey basters). Water samples were then inspected for the presence of immature mosquitoes and stored in plastic containers (100 ml) for transport. All the material collected was transported to the Miami-Dade County Mosquito Control Laboratory, immature mosquitoes were preserved in 70% ethanol and subsequently morphologically identified using taxonomic keys [34].

Biodiversity indices for the collected mosquitoes were calculated based on the Shannon’s diversity index [35] and Simpson’s dominance index [36]. Individual rarefaction curves were generated to estimate both sampling sufficiency and expected occurrence of species for smaller samples. The test requirements for taxonomic proximity of samples, standardized sampling effort and collection in similar habitats were met [37]. Plots of cumulative species abundance (ln S), Shannon’s index (H) and log evenness (ln E) (SHE) profiles were also calculated for the collected immature mosquitoes. Changes in direction of lines indicate ecological heterogeneity of mosquito assembly [38]. All analyses were carried out with 10,000 randomizations without replacement and 95% confidence interval using the Past software (v.3.16) [39].

## Results

**Fig. 2:**
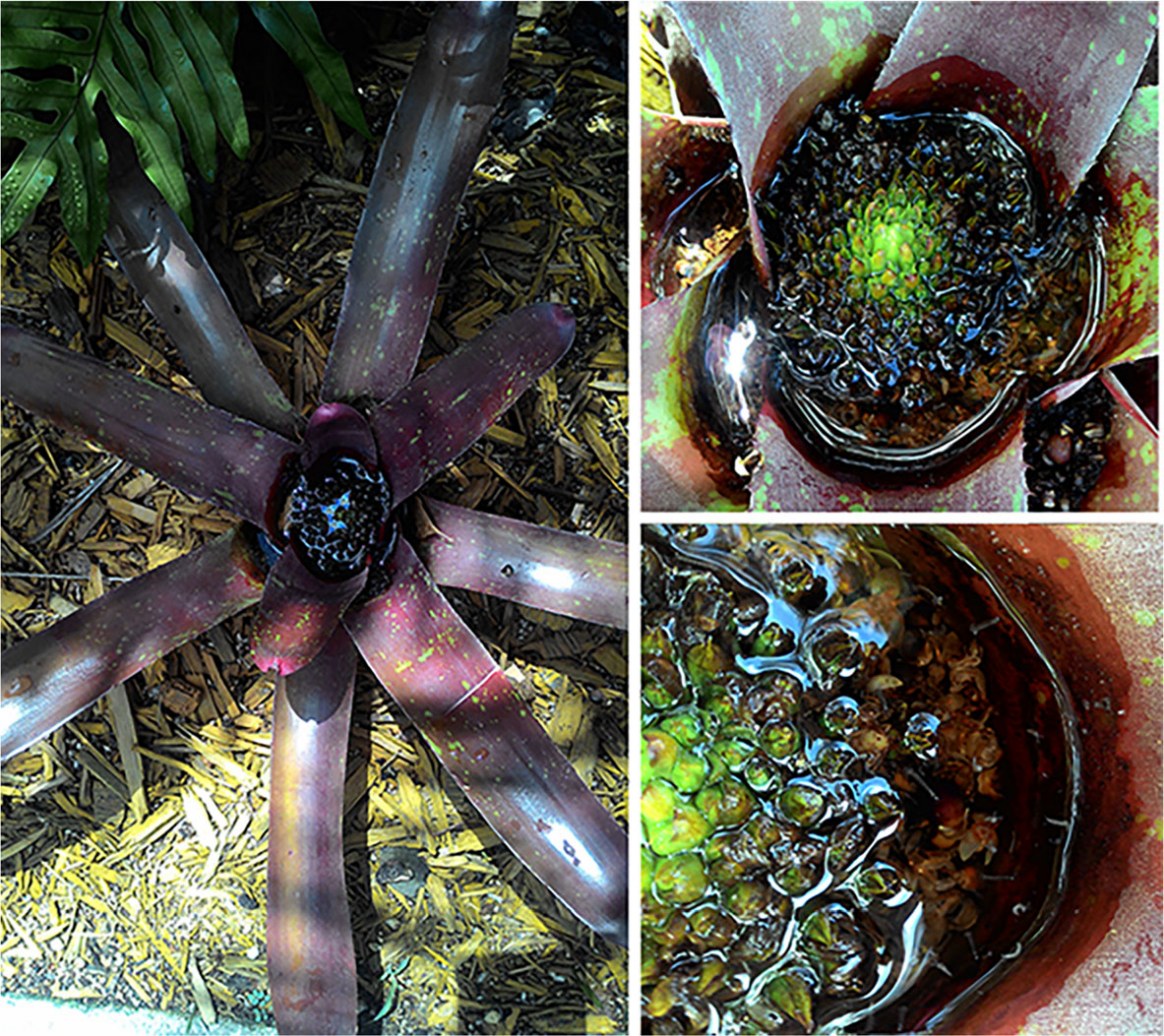
Ornamental bromeliads breeding vector mosquitoes

Ornamental bromeliads were abundantly found at all 51 locations surveyed, being present in residential, commercial and public areas. Moreover, they are popular among landscaping in residential areas, accumulating water and serving as breeding sites for vector mosquitoes (Fig. 2).

A total of 765 immature mosquitoes were collected from ornamental bromeliads water reservoirs, comprising three mosquito genera, *Aedes* (Meigen)*, Culex* (Linnaeus) and *Wyeomyia* (Theobald), and five taxonomic units *Ae. aegypti*, *Wyeomyia mitchellii*, *Wyeomyia vanduzeei*, *Cx. quinquefasciatus* and *Culex biscaynensis.* No predator invertebrates were found preying on immature mosquitoes breeding in ornamental bromeliads. Furthermore, *Ae. aegypti* was the most abundant species found breeding in bromeliads, accounting for around 40% of all specimens collected, followed by *Cx. quinquefasciatus*, *Wy. vanduzeei***,** *Wy. mitchellii* and *Cx. biscaynensis. Aedes aegypti* also had the highest incidence, being collected in 13 from 18 ZIP Code areas surveyed (Table 1).

**Table 1:** Immature mosquito species collected in ornamental bromeliads in the County of Miami-Dade

Neighborhood	Income^a^	ZIP code	*Aedes* (*Ste*.) *aegypti*	*Wyeomyia* (*Wye*.) *mitchellii*	*Wyeomyia* (*Wye*.) *vanduzeei*	*Wyeomyia* sp.	*Culex* (*Cux*.) *quinquefasciatus*	*Culex* (*Cux*.) *biscaynensis*
Hialeah	Low	33010	20	0	108	0	0	0
Hialeah	Low	33012	5	0	4	0	0	0
Hialeah	Low	33013	6	0	6	0	0	0
Wynwood	Low	33127	0	0	0	0	0	0
Coconut Grove	High	33133	36	0	6	0	32	0
Coral Gables	High	33134	57	0	2	0	42	0
Wynwood	Low	33137	17	0	12	0	0	0
South Beach	Medium	33139	9	0	0	0	0	0
Mid Beach	Medium	33140	0	12	0	0	0	0
Lagorce Island	High	33141	1	0	0	1	0	0
Kendall	Medium	33143	28	1	3	0	0	7
Coral Gables	High	33146	45	0	0	0	86	0
Palmetto Bay	High	33157	5	85	8	0	1	6
Coral Gables	High	33158	67	0	2	0	3	6
Miami Springs	Medium	33166	0	11	1	2	0	0
Kendall	Medium	33173	0	2	7	0	0	1
Kendall	Medium	33176	0	0	4	0	0	2
Biscayne Bay	High	33181	5	0	0	0	1	0
			301	111	163	3	165	22

^a^Based on median household income (in 2016 dollars), 2012–2016. US Census Bureau [33]

**Fig. 3:**
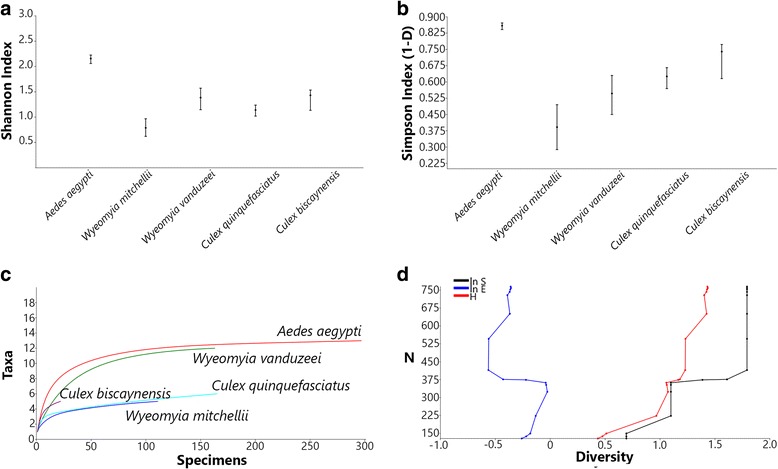
Biodiversity indices for immature mosquitoes collected from ornamental bromeliads in Miami-Dade County. **a** Shannon’s index (H). **b** Simpson’s (1-D) index. **c** Individual rarefaction curves; **d** Plots of cumulative SHE profiles (ln S, H and ln E)

The Shannon’s diversity index had an average of 1.38 (95% CI: 1.19–1.50), indicating low degrees of species diversity in bromeliads (Fig. 3a). Similar results were found for the Simpson’s index, indicating *Ae. aegypti* as the most dominant species breeding in bromeliads, yielding a value of 0.85 (95% CI: 0.84–0.87) (Fig. 3b). The individual rarefaction curves indicated that sampling sufficiency was considerably asymptotic for all species but *Cx. biscaynensis*, with a substantial degree of confidence for predicting the expected presence of those species for smaller sample sizes (Fig 3c). The changes in direction of the lines in the cumulative SHE analysis revealed the heterogeneity of species composition, diversity and evenness for the immature mosquitoes found breeding in bromeliads in different areas (Fig. 3d).

## Discussion

Bromeliads plants embody a complex scenario, in which their importance in breeding vector-mosquitoes may vary according to locality, habitats, vector species, human behavior and climate [23]. Our findings suggest that ornamental bromeliads are contributing for the proliferation of *Ae. aegypti* in the County of Miami-Dade. The analysis revealed a low diversity scenario, in which *Ae. aegypti* is the most dominant species breeding in bromeliads as well as the most likely to be found in these plants. Moreover, notwithstanding the great variation found for the mosquito community structure, *Ae. aegypti* was the least affected species exhibiting a higher incidence and abundance among the species found in bromeliads. In addition, *Aedes albopictus* were not found breeding in ornamental bromeliads; however, this result may be explained by its relatively low occurrence in Miami-Dade County. According to the Miami-Dade Mosquito Control Division, from January to August 2017, 25,946 specimens of *Ae. aegypti* were collected, contrasting with only 380 specimens of *Ae. albopictus* collected during the same period.

The results obtained in this study contrast with previous studies, which do not consider bromeliads as important potential breeding sites for *Ae. aegypti* [26, 28, 40], including for South Florida [41]. However, Miami-Dade County has been undergoing intense efforts to control vector mosquitoes, employing both chemical and biological strategies, as well as breeding site removal as part of IVM strategies, which may translate as strong selective pressures for *Ae. aegypti* populations that may be leading to a shift in their behavior and triggering adaptation processes. The fact that immature *Ae. aegypti* had been more abundantly found breeding in bromeliads than *Wy. mitchellii* and *Wy. vanduzeei*, considered adapted to breed in plants from the family *Bromeliaceae*, with implications to local control strategies. Also, the fact that no predator has been found preying on immature mosquitoes breeding in ornamental bromeliads constitutes a habitat in which vector-mosquitoes can potentially grow indiscriminately, positively driving their abundance.

Nevertheless, our results should be interpreted with caution since vector mosquitoes breeding in bromeliads comprise a complex scenario with wide variation between environments and localities, resulting in the impossibility of transposing our findings for another area besides Miami-Dade County. Additionally, the cross-sectional experimental design without re-sampling chosen for this study may have resulted in the underestimation for the abundance of vector-mosquitoes as well as not having been able to detect rare species breeding in ornamental bromeliads.

In view of the Miami-Dade scenario, there are very few options to prevent ornamental bromeliads from becoming potential breeding sites for vector mosquitoes. Granules or pellets containing the insect growth regulator (S)-methoprene have been successfully used for controlling *Ae. aegypti* immatures in bromeliads [42], commercial larvicides based on toxins extracted from *Bacillus thuringiensis israelensis* (Bti) are also available. However, ornamental bromeliads are highly abundant and the fact that every plant must be treated frequently hampers the process. Flushing the water accumulated in plants reservoirs with a hose is also unlikely to prevent mosquitoes from breeding since it is virtually impossible to reach to every single water reservoir and remove all larvae. Moreover, there is an inherent risk to this scenario due to the use of ornamental bromeliads in landscaping in densely populated areas, where reduced overall biodiversity of urban areas results in fewer availability of hosts for blood-feeding, favoring anthropophilic species of mosquitoes, and consequently increasing the risk of vector-borne diseases.

## Conclusions

The ZIKV outbreak that struck Miami in 2016 created panic among the public, greatly impacting the economy and endangering residents and tourists. The present study provides evidence that the County responded appropriately during the ZIKV outbreak crisis. Our scientific findings exposed an unexpected scenario for Miami-Dade County, in which *Ae. aegypti* immatures are successfully breeding in ornamental bromeliads. To our understanding, it is recommended that during emergency situations control methods should be employed systematically on phytotelmata bromeliads, and removal of plants should be considered when needed.

## Additional file

Additional file 1: **Figure S1.** Miami-Dade County Mosquito Control Division brochure alerting for the risk of ornamental bromeliads as potential breeding sites for vector-mosquitoes. Available in: https://www.miamidade.gov/solidwaste/library/brochures/bromeliads-and-mosquitoes.pdf. (TIF 3033 kb)

## Abbreviations

Bti: *Bacillus thuringiensis israelensis*
CHYKV: Chikungunya virus
DENV: Dengue fever virus
IVM: Integrated vector management
WHO: World Health Organization
YFV: Yellow fever virus
ZIKV: Zika virus
