# Supplementary figures and images for: Ornamental bromeliads of Miami-Dade County, Florida are important breeding sites for Aedes aegypti (Diptera: Culicidae)
Source: Parasit Vectors. 2018 May 17;11:283. doi: 10.1186/s13071-018-2866-9 (PMC5956556; doi:10.1186/s13071-018-2866-9)

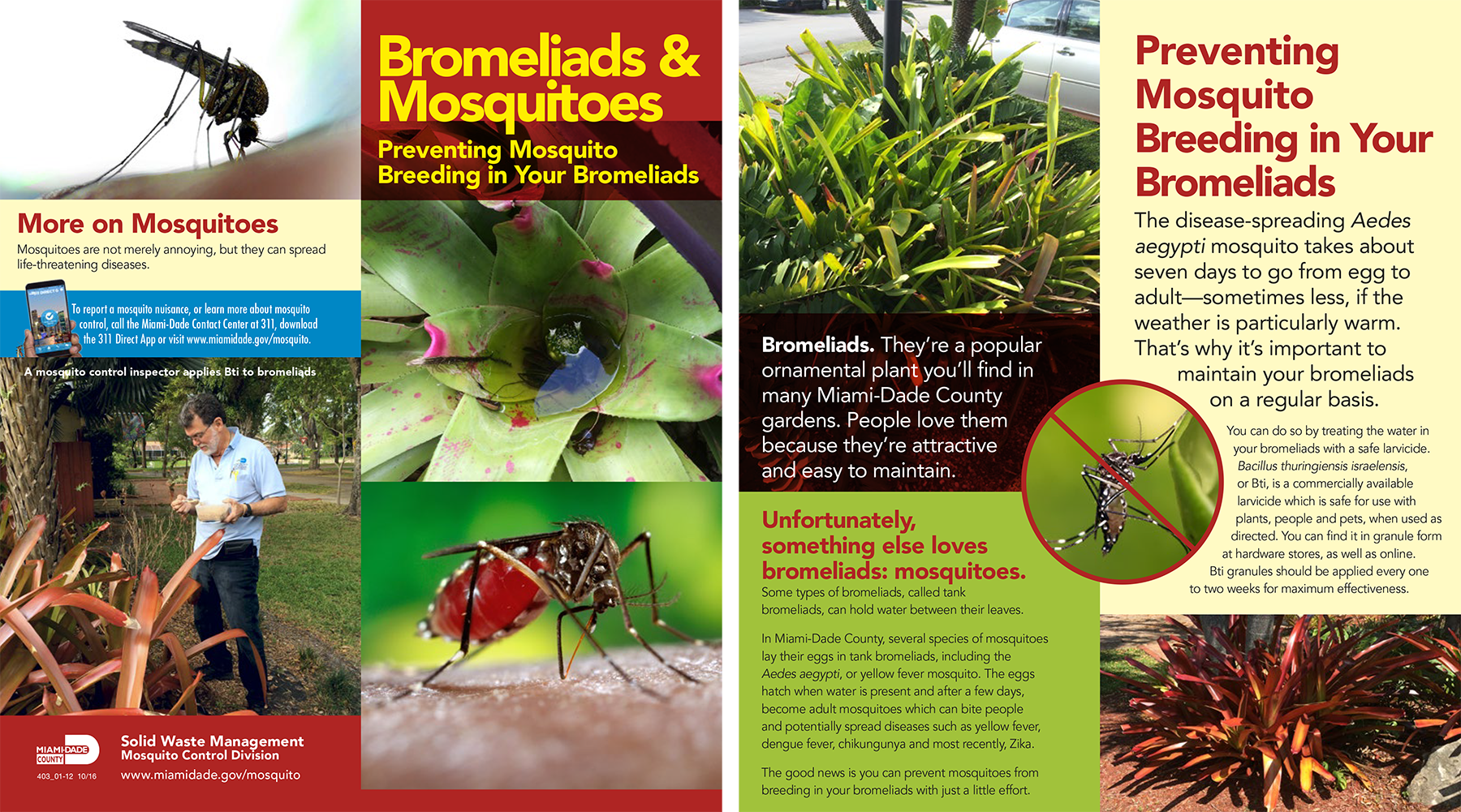

Supplement: Supplementary file 1 — Figure S1. Miami-Dade County Mosquito Control Division brochure alerting for the risk of ornamental bromeliads as potential breeding sites for vector-mosquitoes. Available in: https://www.miamidade.gov/solidwaste/library/brochures/bromeliads-and-mosquitoes.pdf. (TIF 3033 kb) [file 13071_2018_2866_MOESM1_ESM.tif]
